# Pharmacological treatment to reduce pulmonary morbidity after esophagectomy

**DOI:** 10.1002/ags3.12469

**Published:** 2021-07-01

**Authors:** Hiroharu Shinozaki, Tadashi Matsuoka, Soji Ozawa

**Affiliations:** ^1^ Department of Surgery Saiseikai Utsunomiya Hospital Tochigi Japan; ^2^ Department of Gastroenterological Surgery Tokai University School of Medicine Kanagawa Japan

**Keywords:** esophageal cancer, esophagectomy, pharmacological treatment, pulmonary complication

## Abstract

Esophagectomy for esophageal cancer is one of the most invasive procedures in gastrointestinal surgery. An invasive surgical procedure causes postoperative lung injury through the surgical procedure and one‐lung ventilation during anesthesia. Lung injury developed by inflammatory response to surgical insults and oxidative stress is associated with pulmonary morbidity after esophagectomy. Postoperative pulmonary complications negatively affect the long‐term outcomes; therefore, an effort to reduce lung injury improves overall survival after esophagectomy. Although significant evidence has not been established, various pharmacological treatments for reducing lung injury, such as administration of a corticosteroid, neutrophil elastase inhibitor, and vitamins are considered to have efficacy for pulmonary morbidity. In this review we survey the following topics: mediators during the perioperative periods of esophagectomy and the efficacy of pharmacological therapies for patients with esophagectomy on pulmonary complications.

## Graphical abstract

Schema of lung injury during esophagectomy. Lung injury is associated with tissue injury by the surgical procedure and one‐lung ventilation during anesthesia. Colored lines indicate the presumed main action sites of each drug.

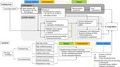

## 1 INTRODUCTION

Esophageal cancer is one of the major causes of cancer mortality worldwide, with more than 473,000 new cases and 436,000 deaths annually.^1^ Esophagectomy for esophageal cancer plays an important role in the strategy for curative treatment, but is associated with considerable morbidity and mortality.^2^, ^3^, ^4^ Morbidity after esophagectomy is significantly correlated with poor prognosis and especially pulmonary and infectious morbidities affected for long‐term outcomes.^5^, ^6^, ^7^, ^8^ Several studies revealed that postoperative pulmonary complications may be an independent predictor of poor long‐term survival in patients undergoing resection of esophageal cancers.^5^, ^6^, ^8^ Among recent advances in the perioperative multidisciplinary treatments for the prevention of pulmonary complication,^9^, ^10^ we focus on pharmacological treatment and discuss immunological mechanisms related to lung injury caused by esophagectomy in this narrative review.

## 2 MEDIATORS DURING THE PERIOPERATIVE PERIODS OF ESOPHAGECTOMY

### 2.1 Lung injury during esophagectomy

**FIGURE 1:**
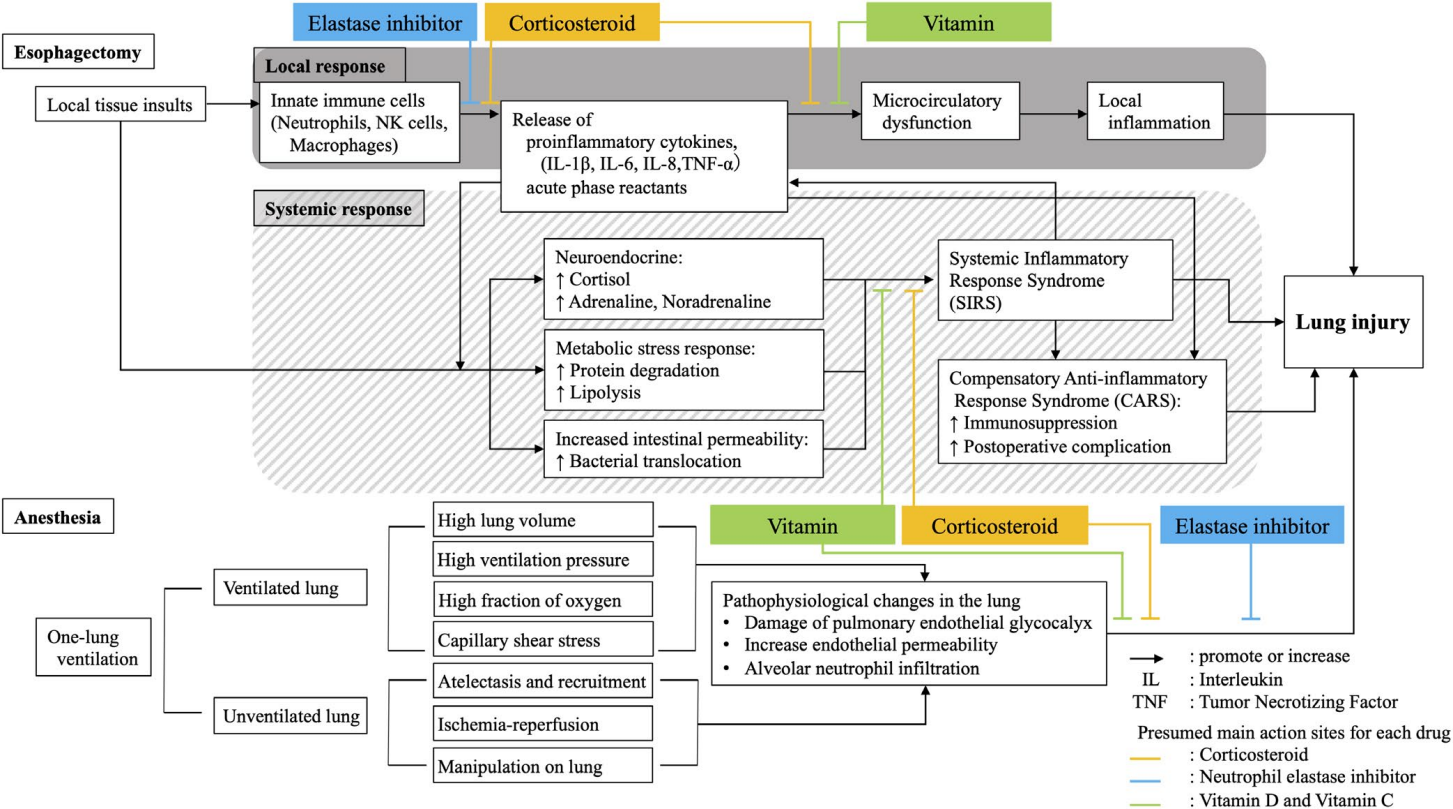
Schema of lung injury during esophagectomy. Colored lines indicate the presumed main action sites of each drug

As one of the most invasive procedures in gastrointestinal surgery, esophagectomy may occasionally cause lung injury postoperatively. Lung injury is associated with tissue injury by the surgical procedure and one‐lung ventilation during anesthesia (Figure 1).^11^ Surgical insults cause alterations in the hemodynamic, metabolic, and immune responses of patients in the perioperative period, resulting in postoperative complications such as pneumonia.^12^ Furthermore, one‐lung ventilation during the operation could damage bilateral lungs, that is, the collapsed lung and the other ventilated lung. In the ventilated lung, high lung volume and ventilating pressure, high fraction of inspired oxygen, and capillary shear stress are related to the development of lung injury.^13^, ^14^, ^15^ On the other hand, in the collapsed lung, atelectasis and recruitment, ischemia‐reperfusion injury, and manipulation on the lung during the operation are considered to be the main causes of lung injury.^16^, ^17^ Not only inflammatory response, but also oxidative stress plays a major role in the development of lung injury following esophagectomy.^11^, ^18^, ^19^, ^20^, ^21^ In the inflammatory and oxidative stress response, the endothelial glycocalyx is considered to represent a common pathway for the lung injury development during one‐lung ventilation, because the endothelial glycocalyx is damaged by most of the recognized lung injurious mechanisms.^22^ This response spreads from local to systemic reaction, which is also known as the systemic inflammatory response syndrome (SIRS). SIRS is followed by a compensatory antiinflammatory response syndrome (CARS) as a sequential reaction, which potentially predisposes the host to septic complications.^23^

### 2.2 Cytokine cascades and pulmonary inflammation

Various cytokine cascades, which consist of a complex biochemical network, are activated in acute response to surgical insults and work with diverse effects after invasive surgery. The invasive procedures such as esophagectomy sometimes cause an exaggerated production of proinflammatory cytokines, which can lead to systemic hemodynamic instability or metabolic derangements.^12^ In particular, tumor necrosis factor‐α (TNF‐α) and interleukin 1β (IL‐1β) are promoted by macrophages and monocytes at the primary local operative site, and play a key role during the acute phase response to surgical insults.^24^ Stimulated by TNF‐α and IL‐1β, this acute phase response is followed by the production and release of interleukin (IL)‐6.^25^ Several previous studies reported that surgical insults produce inflammatory mediators such as IL‐6, IL‐8, high‐mobility group box chromosomal protein‐1 (HMGB‐1), or neutrophil elastase in esophagectomy and the elevation of these mediators was associated with pulmonary inflammation.^26^, ^27^, ^28^

### 2.3 Microcirculatory dysfunction and local inflammation

Higher invasive surgery generally tends to have more intraoperative blood loss and microcirculatory disturbances due to hypoperfusion, which is thought to involve endogenous TNF‐α release.^12^, ^29^ These inflammatory responses are also associated with an increase in microvascular hyperpermeability, which can lead to postoperative complications such as lung injury and cardiovascular dysfunction.^29^ Moreover, microcirculatory disturbances following high invasive surgery are also related to alterations in intestinal permeability, which can lead to postoperative bacterial translocation and can exacerbate surgically induced SIRS and postoperative lung injury following esophagectomy.^11^, ^30^, ^31^

### 2.4 Metabolic alterations

Invasive surgery such as esophagectomy reduces metabolism for ~24 h postoperatively and subsequently causes a catabolic phase.^32^ In the catabolic phase, an increase in endogenous steroid hormone as a counter‐response, induced by TNF‐α and IL–1β, causes inhibition of protein synthesis, increased muscular protein degradation, and mobilization of fats by lipolysis.^33^, ^34^, ^35^ In postoperative metabolic alterations, which occurs frequently in invasive surgery, it is vital to attenuate the catabolic response and support an anabolic phase effectively, which follows a catabolic phase and yields protein synthesis.^36^ Early enteral nutrition reduces the catabolic response to surgical stress and supports an anabolic phase effectively, which can improve surgical outcomes, including pulmonary complications.^36^, ^37^

### 2.5 Innate immune alterations

Although IL‐6 functions as a proinflammatory cytokine in the early postoperative period, it can also exert antiinflammatory effects by attenuating TNF‐α and IL‐1 activity^38^ and inducing macrophages to release prostaglandin E2 and IL‐10,^39^, ^40^ which are central reactions in the CARS in esophagectomy.^41^, ^42^, ^43^, ^44^ Invasive surgery such as esophagectomy also produces alterations in innate immune homeostasis, in which, total systemic CD4+ and CD8+ T‐lymphocyte counts are reduced markedly.^44^, ^45^, ^46^ These immunosuppressed states in the CARS predispose the host to sepsis and are also associated with mortality and significant morbidity, such as postoperative pulmonary complications.^47^, ^48^, ^49^, ^50^, ^51^ Moreover, these states have been associated with an increased rate of tumor progression and metastasis formation in patients with malignant diseases.

### 2.6 Minimally invasive esophagectomy and inflammatory response

Recently, minimally invasive esophagectomy (MIE) such as thoracoscopic, mediastinoscopic, or robot‐assisted esophagectomy is performed more frequently for esophageal cancer treatment. MIE reduces surgical insults, which are related to the reduction of mortality and morbidities.^52^, ^53^ In addition, it is reported that minimally invasive surgery including thoracoscopic and laparoscopic surgery can significantly reduce the rate of tumor progression when compared with open surgery, attenuating suppression of natural killer and lymphokine‐activated killer cell cytotoxicity, which lead to prevention of the accelerated tumor growth.^54^, ^55^

## 3 PHARMACOLOGICAL TREATMENTS

In order to attenuate these acute responses, various treatments based on the pharmacological effect were developed and performed. Here we discuss and review some of the pharmacological treatments to reduce pulmonary complications in esophagectomy for esophageal cancer.

### 3.1 Corticosteroid

Corticosteroid suppresses the production of cytokines such as IL‐6 and TNF‐α in monocytes and macrophages. In esophagectomy, these cytokines are associated with tissue injury from the surgical procedure and one‐lung ventilation further develops postoperative lung injury. Therefore, the presence of a corticosteroid is considered to reduce lung injury by reducing these inflammatory and oxidative responses.^56^ It was reported that corticosteroids had no effect on serum cytokines already released.^57^ Therefore, a corticosteroid was mainly administrated preoperatively or at the induction of anesthesia, which was reported to reduce postoperative IL‐6 levels and the incidence of peripheral leukocytopenia caused by surgical stress.^58^

Based on these theoretical mechanisms, the perioperative corticosteroid for patients with transthoracic esophagectomy was conventionally administered to improve mortality and pulmonary complications in Asia, especially in Japan. To evaluate the efficacy of perioperative corticosteroid, several studies were performed (Table 1).^48^, ^59^, ^60^, ^61^, ^62^, ^63^, ^64^, ^65^ Sato et al pointed out the effect of the preoperative methylprednisolone on the reduction of pulmonary complications.^48^ On the other hand, Takeda et al reported no significant differences in the incidence of pulmonary complications.^65^ As the sample size in each study was relatively small and, due to the lack of consensus, the efficacy of perioperative corticosteroid remains controversial. Engelman et al performed a meta‐analysis from eight randomized control studies (RCTs) and reported that preoperative methylprednisolone usage significantly decreased the incidence of pulmonary complications without an increase of the incidence of other complications such as anastomotic leakage and infections.^64^ On the other hand, Weijs et al performed a meta‐analysis from seven RCTs and reported that there was no significant effect of perioperative methylprednisolone usage on the incidence of pulmonary complications or other complications such as anastomotic leakage and infections.^58^ Based on these nonconclusive results, the Japanese esophageal cancer practice guidelines weakly recommend the clinical use of perioperative corticosteroid administration.^66^, ^67^ In addition, patients in these meta‐analyses were all treated by transthoracic esophagectomy, and not MIE. To date, the efficacy of MIE has been reported and has become increasingly popular.^3^, ^68^, ^69^, ^70^, ^71^, ^72^ MIE is considered to reduce the invasiveness of the operation and decrease postoperative pulmonary complications.^68^, ^69^, ^70^ Several studies evaluating the efficacy of MIE reported a significant reduction in the incidence of pulmonary complications compared to that of open esophagectomy.^71^, ^72^ Based on these reports, the efficacy of the perioperative corticosteroid for patients with MIE may be limited due to the relatively less incidence of pulmonary complication in MIE. To clarify the relationships between the use of the perioperative corticosteroid and MIE, further randomized controlled trials are needed.

**TABLE 1:** Incidence of postoperative pulmonary complications after esophagectomy with or without steroids

Author	Study design	Year	Patient number		Administration of the drug			Pulmonary complications				
			MP group	Control	Dose	Timing	Duration	MP group (%)	Control (%)	Risk ratio	95% CI	*P* value
Yano et al^59^	RCT	2005	20	20	500 mg	Preoperative	120 min	6 (20)	3 (15)	2.00	(0.58–6.91)	.450
Takeda et al^65^	RCT	2003	7	10	10 mg/kg	Before anesthesia	NA	0 (0)	0 (0)	NA	NA	NA
Sato et al^48^	RCT	2002	33	33	10 mg/kg	Preoperative	30 min	3 (13)	10 (30)	0.30	(0.09–0.99)	.061
Takemura et al^60^	RCT	1999	9	9	250 mg	Preoperative	30 min	1 (11)	0 (0)	3.00	(0.14–65.16)	1.000
Matsutani et al^61^	RCT	1998	14	19	10 mg/kg	Before anesthesia	NA	0 (0)	2 (11)	0.27	(0.01–5.15)	.496
Takeda et al^62^	RCT	1997	15	15	30 mg/kg	Before anesthesia	NA	0 (0)	5 (33)	0.09	(0.01–1.51)	.042
Sayama et al^63^	RCT	1994	8	9	250 mg	Preoperative	120–180 min	1 (13)	1 (11)	1.13	(0.08–15.19)	1.000
Engelman et al^64^	Meta‐analysis	210	175	171						0.41	(0.24–0.71)	
Weiji et al^58^	Meta‐analysis	2014	281	286						0.69	(0.26–1.79)	

Abbreviations: MP, methylprednisolone; NA, not available; RCT, randomized control trial.

### 3.2 Neutrophil elastase inhibitor

Sivelestat sodium hydrate, a synthetic neutrophil elastase inhibitor, can competitively inhibit neutrophil elastase activity enhanced by IL‐8 during surgical stress. This synthetic neutrophil elastase inhibitor is thought to be related to the increase in lung vascular permeability.^73^, ^74^, ^75^ In addition, sivelestat sodium hydrate suppresses proinflammatory cytokines, including IL‐1β and IL‐6, similar to the mechanisms of corticosteroids.^28^

These antiinflammatory mechanisms are considered to improve postoperative lung injury in esophagectomy. Sivelestat sodium hydrate may be considered for clinical use to attenuate lung injury after esophagectomy,^26^ but its use is not approved by insurance in Japan. Although several studies have shown the therapeutic effect of a neutrophil elastase inhibitor, its efficacy in a clinical setting remains controversial (Table 2).^27^, ^28^, ^76^, ^77^, ^78^, ^79^, ^80^, ^81^ Kuwahara et al reported that perioperative administration of neutrophil elastase inhibitor at the beginning of surgery did not shorten the duration of mechanical ventilation after thoracoscopic esophagectomy (median [h] interquartile range [IQR], 24.5 [24.3–28.7] vs 24.5 [23.9–49.1], *P* = .796).^27^ On the other hand, Makino et al evaluated the efficacy of neutrophil elastase inhibitor in an RCT and reported that perioperative neutrophil elastase inhibitor at the beginning of surgery maintained postoperative pulmonary function following video‐assisted esophagectomy and shortened the duration of mechanical ventilation (median [h] [IQR], 89.5 [57.3–121.7] vs 204 [77.4–330.6], *P* = .046).^76^ Suda et al also reported that perioperative administration of neutrophil elastase inhibitor, which is intravenously administered immediately after surgery, shortened the duration of mechanical ventilation after transthoracic esophagectomy (median [day] [IQR], 1.5 [1–1.5] vs 2 [1.5–2], *P* = .008).^28^ Due to the limited sample size and conflicting conclusion from these studies, Wang et al performed a systematic review and meta‐analysis to evaluate the benefit and safety of neutrophil elastase inhibitor administration. This meta‐analysis, including data from nine studies, revealed that neutrophil elastase inhibitor administration significantly decreased the duration of mechanical ventilation and the incidence of acute lung injury.^82^ The administration of the neutrophil elastase inhibitor did not decrease the incidence of pneumonia, intensive care unit stay, or postoperative hospital stay. However, this meta‐analysis included trials of non‐RCTs and had dissimilar procedures, such as minimally invasive or traditional surgery, which could affect patient outcomes. In addition, the combined effect of corticosteroids and neutrophil elastase inhibitors was not evaluated. Therefore, multi‐institutional RCTs with a large sample size are needed to validate the efficacy of perioperative neutrophil elastase inhibitor administration for esophagectomy.

**TABLE 2:** Incidence of postoperative pulmonary complications after esophagectomy with or without neutrophil elastase inhibitor

Author	Study design	Year	Surgical approach	Adjunct therapy	Patients number		Administration of the drug			Pulmonary complications				
					EI Group	Control	Dose	Timing	Duration	EI Group	Control	Risk ratio	95%CI	P value
^a^Kawahara et al^27^	RCT	2010	MIE	MP	10	10	300 mg/d	Beginning of surgery	72 h	2 (20%)	2 (20%)	1.00	(0.17–5.77)	1.000
^a^Makino et al^76^	RCT	2011	MIE	None	16	15	4.8 mg/kg/d	Beginning of surgery	7 d	4 (25%)	3 (20%)	1.25	(0.33–4.68)	1.000
^a^Ono et al^77^	Non–RCT	2007	Thoracotomy	None	7	10	4.8 mg/kg/d	ICU admission	6 d	0 (0%)	7 (70%)	0.09	(0.01–1.35)	0.004
^a^Mimatsu et al ^78^	Non‐RCT	2011	Thoracotomy	NA	22	20	4.8 mg/kg/d	ICU admission	5 d	NA	NA	1.21	(0.31–4.77)	NA
^a^Iwahashi et al^79^	Non‐RCT	2011	Thoracotomy	MP	15/15	15	4.8 mg/kg/d	Closure of the chest	2 d/5 d	0 (0%)	1 (1%)	0.33	(0.01–7.58)	0.333
^a^Nishiyama et al^80^	Non‐RCT	2012	Thoracotomy	None	26	27	4.8 mg/kg/d	Beginning of surgery	3 d	3 (10%)	4 (16%)	0.78	(0.19–3.15)	1.000
^a^Nagai et al^81^	Non‐RCT	2013	Thoracotomy	MP	42	35	4.8 mg/kg/d	Beginning of surgery	3 d	5 (12%)	4 (11%)	1.04	(0.30–3.59)	1.000
^a^Wang,et al^82^	Meta‐analysis	2015			153	147						0.84	(0.47–1.50)	
^b^Suda et al^28^	RCT	2007	Thoracotomy	None	18	25	4.8 mg/kg/d	Immediately after surgery	6 d	0 (0%)	1 (0%)	0.47	(0.02–11.01)	1.000
^b^Makino et al^76^	RCT	2011	MIE	None	16	15	4.8 mg/kg/d	Beginning of surgery	7 d	2 (13%)	8 (53%)	0.23	(0.06–0.93)	0.023
^b^Wang,et al^82^	Meta‐analysis	2015			34	40						0.27	(0.08–0.93)	

Abbreviations: EI, neutrophil elastase inhibitor; MIE, minimally invasive esophagectomy; MP, methylprednisolone; NA, not available; RCT, randomized control trial.

^a^ Pneumonia was evaluated as a pulmonary complication.

^b^ Acute lung injury was evaluated as a pulmonary complication.

### 3.3 Vitamins

Some vitamins have cell‐protective effects. Vitamin D, which is a regulator of nitric oxide synthesis in endothelial cells, enhances the activity of antioxidative enzymes attenuating reactive oxygen species (ROS), and inhibits production of proinflammatory mediators such as TNF‐α and IL‐6 by suppressing nuclear factor kappa B (NF‐κB) signaling. These protective effects of vitamin D are considered to protect the pulmonary endothelium^83^ and are considered to reduce postoperative lung injury. Vitamin C also attenuates inflammatory cytokines such as TNF‐α and IL‐6. Vitamin C also reduces oxidative stress by scavenging free radicals, preventing further damage to the alveolar capillaries. Vitamin C inhibits the activation of neutrophil, resulting in the increase of alveolar fluid clearance and preventing lung vascular endothelial injury.^84^ Studies have shown that these effects are synergically enhanced by the presence of corticosteroids.^84^, ^85^

A couple of studies evaluated the efficacy of several vitamin therapies, including trials using vitamins D and C (Table 3). Parekh et al conducted a randomized, placebo‐controlled trial to evaluate the efficacy of vitamin D for patients with open esophagectomy or MIE.^86^ In this study, a single oral preoperative vitamin D administration did not improve postoperative lung function. On the other hand, recent studies reported that vitamin C was applied to various conditions such as septic shock and acute respiratory distress syndrome (ARDS).^84^, ^85^, ^87^ We have reported the therapeutic effect of the combination therapy of vitamin C and corticosteroid on postoperative lung function after esophagectomy.^88^ However, this study was a single‐center retrospective study and a before‐after study with a small sample size. Therefore, additional large‐scale prospective studies are required.

**TABLE 3:** Incidence of postoperative pulmonary complications after esophagectomy with or without vitamins

Drug	Study design	Year	Surgical approach	Adjunct therapy	Patients number		Administration of the drug			Pulmonary complications					P/F ratio improvement
Author					Vit. Group	Control	Dose	Timing	frequency	Vit. Group	Control	Risk ratio	95% CI	*P* value	
Vit. D															
Parekh et al^86^	RCT	2018	Thoracotomy	None	33	35	7.5 mg	Preopeartive	Single dose	17 (52%)	17 (49%)	1.06	(0.66–1.71)	1.000	No
Vit. C															
Matsuoka et al^88^	Non‐RCT	2019	Thoracoscopy	MP	6	11	1.5 g	Beginning of surgery	Every 6 h for 4 d	2 (33%)	5 (45%)	0.73	(0.20–2.70)	1.000	Yes

Abbreviations: MP, methylprednisolone; RCT, randomized control trial; Vit., vitamin.

### 3.4 Others

Drugs that suppress the production of inflammatory mediators, such as cytokines or antioxidant agents that have cell‐protective effects have the possibility of reducing pulmonary morbidity by attenuating lung injury during invasive surgery, theoretically. However, few drugs other than steroids, vitamin C, vitamin D, and neutrophil elastase inhibitor have been reported to be clinically effective. In this context, gas inhalation therapy might have the potential to reduce pulmonary morbidity after esophagectomy. β‐agonist inhalation effectively treats ARDS through decreasing inflammatory cell infiltration and cytokine release, augmenting alveolar fluid clearance, and improving alveolar capillary barrier function.^89^ However, Perkins et al reported that there is no significant difference in the incidence of ARDS in postoperative patients with esophagectomy between β‐agonists, salmeterol, and placebo (odds ratio [OR], 1.25; 95% confidence interval [CI], 0.71–2.22).^90^ Inhaled nitric oxide, a well‐known inhalant for the treatment of lung injury, was considered to decrease pulmonary artery pressure and improve oxygenation, but has not shown efficacy for ARDS. The specific therapeutic effects of nitric oxide inhalation for postoperative lung injury in esophagectomy remains unclear, as no study targeted the population of patients with esophagectomy.^91^ These inhalation therapies need further investigation to be considered one of the mainstream treatment options.

## 4 CONCLUSION

Lung injury following esophagectomy is associated with an invasive surgical procedure and one‐lung ventilation during anesthesia. Surgical insults cause alterations in hemodynamic, metabolic, and immune responses, and one‐lung ventilation damages bilateral lungs by inflammatory and oxidative stress responses. Local response to tissue insults spreads to a systemic response through inflammatory mediators resulting in lung injury. Some of the pharmacological treatments for preventing lung injury have the potential to improve pulmonary morbidity after esophagectomy. However, further prospective studies with large samples are needed to confirm the efficacy of those therapies to establish better perioperative management for esophagectomy.

## DISCLOSURE

Funding: There was no funding for this review.

Conflict of Interest: The authors declare no conflicts of interest for this article.

Author Contribution: Hiroharu Shinozaki and Soji Ozawa devised the main conceptual ideas for this article. Hiroharu Shinozaki and Tadashi Matsuoka selected and reviewed references and wrote the initial draft of the manuscript. All authors critically reviewed the manuscript.
